# Knowledge, attitude and practice towards child adoption amongst women attending infertility clinics in Lagos State, Nigeria

**DOI:** 10.4102/phcfm.v3i1.259

**Published:** 2011-10-11

**Authors:** Adenike O. Omosun, Odeyemi Kofoworola

**Affiliations:** 1 Department of Community Health and Primary Care, Lagos University Teaching Hospital Idi-araba, Lagos, Nigeria

## Abstract

**Background:**

Child adoption is a recommended alternative form of infertility management. Infertility is of public health importance in Nigeria and many other developing nations. This is a result of its high prevalence and especially because of its serious social implications as the African society places a passionate premium on procreation in any family setting.

**Objectives:**

The aim of this study was to determine the knowledge, attitude and practice of child adoption amongst women attending infertility clinics in teaching hospitals in Lagos State and to determine the factors that influence their attitude and practice towards it.

**Method:**

A cross-sectional descriptive design was used. Data were collected by using a structured questionnaire which was interviewer-administered. The study was conducted in the two teaching hospitals in Lagos State (LUTH [Lagos University Teaching Hospital] and LASUTH [Lagos State University Teaching Hospital]) from amongst 350 women attending the gynaecological clinics. All the patients under management for infertility at the gynaecology clinics during the period of the study were interviewed.

**Results:**

Many respondents (85.7%) had heard of child adoption and 59.3% of them knew the correct meaning of the term. More than half of the respondents (68.3%) said that they could love an adopted child but less than half of them (33.7%) were willing to consider adoption. Only 13.9% has ever adopted a child. The major reason given for their unwillingness to adopt was their desire to have their own biological child. Factors that were favourable towards child adoption were Igbo tribe identity, an age above 40 years, duration of infertility above 15 years, and knowing the correct meaning of child adoption.

**Conclusion:**

There is a poor attitude to adoption even amongst infertile couples. Interventions need to be implemented to educate the public on child adoption, to improve their attitude towards adoption and to make it more acceptable.

## Introduction

Adoption is the act of legally placing a child with a parent or parents other than those to whom they were born. An adoption order has the effect of severing parental responsibilities and the rights of the original parent(s), and transferring those responsibilities and rights to the adoptive parent(s). Adoption can either be an open or a fully disclosed adoption. It allows identifying information to be communicated between adoptive and biological parents and perhaps, interaction between kin and the adopted person. The adoption can also be closed which bars all identifying information from being shared between the adoptive parents, biological kin, and the adoptee.^1^

Ancient adoption practices differed markedly from the modern practices of adoption. Foremost, they were legal tools to strengthen political ties between wealthy families and they provided male heirs to manage the estates of other notables. Adoption was inherently an act that fostered the interests of adults and placed less emphasis on the interests of those adopted. The use of adoption along these lines by the aristocracy is well documented; many of Rome's emperors were adopted sons.^2^ Moreover, evidence suggests that although legal, infant adoption for the purpose of building a family was rare. The abandoned were often rounded up for slavery rather than for adoption purposes.^3^

In Lagos State, Nigeria, there are three types of adoption, namely local (within the country), international (outside the country) and relative adoption within a family. A common example of this is a ‘stepparent adoption’, where the new partner of a parent may legally adopt a child from the parent's previous relationship. Intra-family adoption can also occur through surrender, as a result of parental death, or when the child cannot otherwise be cared for and a family member agrees to assume responsibility.^4^

Today, however, infertility is the main reason parents seek to adopt children to whom they are not related.^4^ Other motivations for adoption could be a desire to provide a home to a homeless child, to gain a child of the other sex, advanced age and the possibility of genetic problems in the person's biological child.

Infertility is of public health importance in Nigeria and many parts of Africa because of its high prevalence ranging from 10% – 20% in sub-Saharan Africa^5^ and as high as 32% in Gabon, especially as a result of its serious social implications. The African society places extreme emphasis on procreation in any family setting. The woman's place in marriage remains precarious until it is confirmed through child bearing. In society a woman has to prove her womanhood through motherhood. A man has to confirm his manhood in the same fashion. Children are esteemed as sources of pride, strength and economic fortune for the family, with a man's wealth and strength being equated to his progeny.^6^

In general women are held responsible for virtually all cases of infertility. Menfolk are seen as blameless. Consequently the woman is humiliated, isolated, derided, abused and rebuffed. Such life crises have been the experience of most infertile women in Africa. Amongst the Yoruba tribe in Nigeria, infertile women are called ‘*agon*’, from a word that means ‘to hold in contempt or to despise’.7 Even when male infertility is the reason a couple cannot have children, women may still face the threat of divorce.^7^ They go to varying lengths, therefore, to visit orthodox medical practitioners, herbalists, traditionalists and spiritualists in search of the needed reprieve and a solution. That is why some women apply various strategies to have children such as visiting traditional healers and prayer houses. They would even buy babies (following the faking of their pregnancy) without caring about the source, or engage in secret extramarital sexual relationships.^6^ Others who can afford it, try assisted reproductive techniques which are very expensive and have a low success rate.

### Setting

In Lagos State, child adoption is organised via the Social Welfare arm of the Ministry of Youth who regulates the affairs of abandoned children in the orphanages and motherless babies’ homes under the Child Right Law passed in May 2007.^8^ Child adoption has been on the increase with the recent statistic of 210 children adopted between March 2007 and December 2008 via the ministry, and because of the long waiting list for prospective adopters in the orphanages. There are registered orphanages from which people adopt.^4^

### Key focus

There are not many studies carried out on child adoption in Nigeria, especially in Lagos State. This study therefore aims at providing background information concerning knowledge, attitude and practice towards child adoption amongst patients attending infertility clinics in teaching hospitals in Lagos State namely Lagos University Teaching Hospital (LUTH) and Lagos State University Teaching Hospital (LASUTH) (State tertiary hospital), findings of which can be used to design relevant intervention programmes.

### Significance of the study

It is estimated that between 8% and 12% of couples world wide suffer from infertility. In Africa, this rate may be as high as 30%. The psychological and emotional stress is the worst part, particularly for women in many developing countries who are generally blamed for infertility. Furthermore, limited treatment options are available here, and they are expensive with low success rates, and fewer opportunities for personal fulfilment outside the family exist. Child adoption is an alternative treatment option, but limited studies only have been conducted in this region to provide background information in order to plan interventions to promote it.

## Ethical considerations

Permission was obtained from the Heads of the Department of Obstetrics and Gynaecology in the two teaching hospitals. Written informed consent was also obtained from each of the participants before the questionnaire was implemented.

## Methods

### Materials

The study subjects who were recruited, were women who complained of an inability to conceive after one year of regular sexual intercourse or who were under management for infertility. Both old and new patients were therefore interviewed. Those with a secondary infertility with or without a live-born biological child were also included.

The data collection tool used was a closed ended questionnaire which was interviewer-administered. A doctor in each of the clinics attended to cases of infertility caused by other gynaecological complaints before the clinic started; trained interviewers then presented the questionnaire to the identified participants.

### Context of the study

The study is a descriptive cross-sectional study of women attending infertility clinics in the two teaching hospitals in Lagos state, Nigeria, namely LASUTH and LUTH.

A sample size of 350 participants, obtained by using the formula:

$$N=Z^{2}p(q/d^{2})$$

for sample size estimation,^9^ was used.

All patients who attended the clinics for infertility treatment were recruited consecutively on every clinic day until the required sample size was achieved. Data was collected from 04 May 2009 to 03 June 2009.

### Design

A pre-test was carried out at the Federal Medical Centre, Ebute-metta, which was analysed and corrected before the study commenced.

The questionnaire was divided into five sections:

- Section A referred to the socio-demographic characteristics of the clients.
- Section B questions related to infertility.
- Section C questions assessed knowledge of child adoption.
- Section D questions assessed the attitude of the participants.
- Section E questions assessed their practice.

### Analysing

All data collected were analysed using the Epi Info (version 6.04) statistical software package.^10^ The Chi-squared test and Fisher's exact test was used to measure association. Statistical significance was set at 0.05.

Informed consent was obtained from each of the participants before the questionnaires were presented.

## Results

A total of 350 questionnaires were analysed. The mean age of the respondents was 33.6 ± 4.9 years. Most of the respondents were of the Christian faith (77.4%). The educational level of the respondents was mainly on a tertiary level (49.4%), closely followed by secondary education (42.3%) and a small percentage (2.3%) had no formal education. Most of the respondents were from the ethnic groups of Yoruba (54.6%) and from the Igbo (23.1%); Hausas made up 2.6% and Others constituted 19.7%. All the respondents were married with most of the respondents practicing monogamy (89.4%) (Table 1).

**TABLE 1:** Socio-demographic distribution of respondents (*N* = 350).

Socio-demographic variable	Frequency	%
**Age (years)**		
22–29	66	18.9
30–39	241	68.9
40–49	43	12.2
**Total**	**350**	**100.0**
**Religion**		
Christianity	271	77.4
Islam	79	22.6
**Total**	**350**	**100.0**
**Education**		
No formal education	8	2.3
Primary	21	6.0
Secondary	148	42.3
Tertiary	173	49.4
**Total**	**350**	**100.0**
**Ethnicity**		
Igbo	81	23.1
Yoruba	191	54.6
Hausa	9	2.6
Others	69	19.7
**Total**	**350**	**100.0**
**Marriage type**		
Monogamy	313	89.4
Polygamy	37	10.6
**Total**	**350**	**100.0**

*Source*: Authors’ original data *N*, given as means of number.

The mean duration of infertility was 5.4 ± 4.4 years. The cause of infertility was mainly related to the wife (44.3%), with 9.2% related to the husband and 11.7% to the couple. At the time of the interview, 33.4% did not know the cause and in 1.4% of the respondents, there was no known cause for the infertility (Table 2).

**TABLE 2:** Distribution of respondents by duration and cause of infertility (*N* = 350).

Variable	Frequency	%
**Duration of infertility (years)**		
1–5	229	65.4
6–10	78	22.3
11–15	32	9.1
> 15	11	3.1
**Total**	**350**	**100.0**
**Cause of infertility**		
Husband	32	9.2
Wife	155	44.3
Both	41	11.7
None	5	1.4
Do not know	117	33.4
**Total**	**350**	**100.0**

*Source*: Authors’ original data *N*, given as means of number.

Most of the respondents (74.9%) had taken some form of drug and only 0.6% had tried either artificial insemination or in-vitro fertilisation; 38.3% had taken herbal treatments and 17.1% had surgical treatment.

Most of the respondents (69.1%) have been pregnant, with only 30.9% who have never been pregnant. Amongst those who *have* been pregnant, only 27.7% have a child or children. Amongst those who have children, 80.6% have one child, 17.9% have two children and 1.5% has more than two children.

Of the 350 respondents interviewed, 85.7% have heard of child adoption. The first major source of information about child adoption was the media (40.4%), closely followed by friends (32.3%). Other sources included the church and people employed at orphanages.

Over half of the respondents (59.3%) knew the correct meaning of child adoption as a legal process of taking the child of another as one's own whilst 15.7% thought that child adoption meant buying a motherless baby.

Few of the respondents possessed knowledge of the requirements for application to adopt a child. These requirements include the presentation of a marriage certificate (24.3%), a birth certificate (19.0%), a medical certificate of fitness (23.7%) and passport photographs (20.0%). The majority of the respondents (60.7%) did not know the maximum age of children who may be adopted, 12.0% assumed that there was no maximum age and only 12.3 knew that the maximum age was 18 years.

A significant number (77%) of respondents knew that children who were available for adoption were mostly abandoned or neglected children, whilst 18.3% did not know who could be adopted.

More than half of the respondents did not know the age requirements for people who wanted to adopt a child:

- 42.7% knew a person must be aged 25 or more if married before she would be allowed to adopt
- 34% knew that a person must be at least 21 years older than the child she wanted to adopt
- 30.5% knew that if the person is not married, she must be older than 35.

More than half of the respondents (68.3%) said that they could love an adopted child but only 33.7% were willing to consider adoption and only 9.7% of their spouses had considered adoption (Table 3). The major reason given by the respondents for their unwillingness to adopt a child was, ‘*I want my biological child*’ (82.6%). Other reasons included ’*it is not a solution to my infertility*’, ‘*fear of abnormal child*’ and ‘*psychologically unacceptable*’.

**TABLE 3:** Distribution of respondents by their attitude to child adoption.

Attitude to child adoption	Yes		No		Do not know		Total	
				
	*f*	%	*f*	%	*f*	%	*f*	%
Can love adopted child like biological child	205	68.3	63	21.0	32	10.7	300	100
Willing to consider adopting a child	101	33.7	195	65.0	4	1.3	300	100
Husband has considered adoption	29	9.7	270	90.0	1	0.3	300	100

*Source:* Authors’ original data *f*, frequency.

Of those willing to adopt a child, 54.5% would do so to rescue a child in an irreversible situation of abandonment and only 36.6% would adopt to ease the urge to care for a child. The preferred age for children to be adopted was new-born, that is, younger than six months (46.5%), and six months to two years (35.5%). Most of the respondents willing to adopt did have any particular sex preference (68.3%); however, a slight majority of the respondents would prefer to adopt a girl (18.8%) as opposed to a boy (12.9%). Of those willing to consider child adoption, only 13.9% have ever adopted or attempted to adopt a child.

Just more than half of the respondents willing to adopt (55.4%) felt that children should be told that they were adopted, whilst 44.6% felt that they should not be told. Most of the respondents (64.3%) felt that children should be told that they were adopted only when they attain adulthood. The majority of the respondents who felt children should be told they were adopted were willing to assist the child to find his or her biological relatives and only 2.4% were not sure whether they could do that.

There was no statistically significant association between marriage type, age, religion, the duration of infertility and respondents’ willingness to adopt (*p* > 0.05). There was however, a statistically significant association between ethnicity, educational level and respondents’ willingness to adopt (*p* < 0.05). Igbos were more willing to adopt than Yorubas and women of a higher educational level were more willing to adopt than women of a lower educational level (Table 4).

**TABLE 4:** Associations between socio-demographic characteristics of respondents and their willingness to consider child adoption.

Socio-demographic variables	Willingness to consider adopting a child								*X^2^*	*df*	*p*-value

	Willing		Not willing		Do not know		Total				
			
	*n*	%	*n*	%	*n*	%	*N*	%			
**Age (years)**											
22–29	18	33.3	34	63.0	2	3.7	54	100	8.24	4	0.08
30–39	62	30.5	139	68.5	2	1.0	203	100			
40–49	21	48.8	22	51.2	-	-	43	100			
**Total**	**101**	**33.7**	**195**	**5.0**	**4**	**1.3**	**300**	**100**			
**Marriage type**											
Monogamous	96	35.3	172	63.2	4	1.5	272	100	4.10	2	0.13
Polygamous	5	17.9	23	82.1	-	-	28	100			0.12*
**Total**	**101**	**33.7**	**195**	**65.0**	**4**	**1.3**	**300**	**100**			
**Ethnicity**											
Igbo	34	43.0	42	53.2	3	3.8	79	100	11.18	4	0.03
Yoruba	43	28.1	109	71.2	1	0.7	153	100			
Others	24	35.3	44	64.7	-	-	68	100			
**Total**	**101**	**33.7**	**195**	**65.0**	**4**	**1.3**	**300**	**100**			
**Religion**											
Christianity	88	35.6	156	63.2	3	1.2	247	100	2.48	2	0.29
Islam	13	24.5	39	73.6	1	1.9	53	100			0.26*
**Total**	**101**	**33.7**	**195**	**65.0**	**4**	**1.3**	**300**	**100**			
**Education**											
No formal education	1	14.3	6	85.7	-	-	7	100	26.74	6	0.0002
Primary	4	36.4	7	63.6	-	-	11	100			
Secondary	20	17.4	92	80.0	3	2.6	115	100			
Tertiary	76	45.5	90	53.9	1	0.6	167	100			
**Total**	**101**	**33.7**	**195**	**65.0**	**4**	**1.3**	**300**	**100**			
**Duration of infertility (years)**											
1–5	58	30.1	131	67.9	4	2.1	193	100	9.40	6	0.1522
6–10	22	32.8	45	67.2	-	-	67	100			
11–15	15	51.7	14	48.3	-	-	29	100			
> 15	6	54.5	5	45.5	-	-	11	100			
**Total**	**101**	**33.7**	**195**	**65.0**	**4**	**1.3**	**300**	**100**			

*Source*: Authors’ original data *n, N*, given as a means of number; *X*^2^, Chi-square; *df*, degrees of freedom; *p*-value, statistical significance.

* Fisher's Exact

The association between spousal consideration and respondents’ willingness to consider adoption was statistically significant (*p* < 0.05). Respondents, whose spouses considered adoption, were more willing to consider it as well.

The association between the meaning of child adoption and respondents’ willingness to consider adoption was statistically significant (*p* < 0.05). Respondents who knew the correct meaning of child adoption were more willing to consider adoption.

There was a statistically significant association between respondents’ age, the duration of infertility and their practice of child adoption (*p* < 0.05). Women of an older age group (40–48 years) with a longer duration of infertility (more than 15 years) were more likely to adopt (Table 5).

**TABLE 5:** Associations between socio-demographic characteristics of respondents and their practice of child adoption.

variable	Practice of child adoption						*X^2^*	*df*	*p*-value

	Ever adopted		Never adopted		Total				
		
	*n*	%	*n*	%	*N*	%			
**Age (years)**									
22–29	-	-	18	-	18	100	9.91	2	0.01
30–39	7	11.3	55	88.7	62	100			0.01*
40–49	7	33.3	14	66.7	21	100			
**Total**	**14**	**13.9**	**87**	**86.1**	**101**	**100**			
**Marriage type**									
Monogamous	13	13.5	83	86.5	96	100	0.07	1	0.80
Polygamous	1	20.0	4	80.0	5	100			0.53*
**Total**	**14**	**13.9**	**87**	**86.1**	**101**	**100**			
**Ethnicity**									
Igbo	2	5.9	32	94.1	34	100	6.70	2	0.04
Yoruba	5	11.6	38	88.4	43	100			0.05*
Others	7	29.2	17	70.8	24	100			
**Total**	**14**	**13.9**	**87**	**86.1**	**101**	**100**			
**Religion**									
Christianity	11	12.5	77	87.5	88	100	0.36	1	0.55
Islam	3	23.1	10	76.9	13	100			0.26*
**Total**	**14**	**13.9**	**87**	**86.1**	**101**	**100**			
**Education**									
No formal education	-	-	1	100	1	100	0.77	3	0.86
Primary	-	-	4	100	4	100			0.80*
Secondary	2	10.0	18	90.0	20	100			
Tertiary	12	15.8	64	84.2	76	100			
**Total**	**14**	**13.9**	**87**	**86.1**	**101**	**100**			
**Duration of infertility (years)**									
1–5	-	5.2	55	94.8	58	100	10.22	3	0.02
6–10	-	22.7	17	77.3	22	100			0.01*
11–15	-	20.0	12	80.0	15	100			
> 15	-	50.0	3	50.0	6	100			
**Total**	**-**	**13.9**	**87**	**86.1**	**101**	**100**			

*Source*: Authors’ original data *n, N*, given as a means of number; *X^2^*, Chi- square; *df*, degrees of freedom; *p*-value, statistical significance.

* Fisher's Exact

Marriage type, religion and education were not significantly related to their practice (*p* > 0.05). The association between respondents’ interpretation of child adoption and their practice of child adoption was not statistically significant (*p* > 0.05).

## Discussion

The modal age of respondents was the age group 30–39. Most of the respondents were Christians (77.4%) The educational level of the respondents was mainly on a tertiary level (49.4%), closely followed by secondary education (42.3%); however, 2.3% had no formal education. This is similar to a study conducted in the South-east of Nigeria amongst infertile women where the modal age was also within the age group of 30–39; 82.5% of the respondents had attained tertiary and secondary education with only 1.4% who had no formal education, showing a similar trend in the age at which infertility clinics were attended and the educational level.11 The ethnicity of most of the respondents was Yoruba, followed by the Igbo. This is expected as the study was conducted in the South-west which is mainly a Yoruba-speaking area. Most of the respondents practice monogamy (89.4%). In African society, a woman's place in marriage is precarious until confirmed through child bearing and a man usually marries a second wife in an attempt to have a child.^12^

The mean duration of infertility was 5.4 ± 4.4 years. This is similar to another study where the mean duration of infertility amongst women attending infertility clinics was 5.0 ± 3.1 years.^11^ In this study, the cause of infertility was mainly attributed to female factors (44.3%). The husband accounted for 9.2% and the couple for 11.7% of cases; 33.4% were yet to be diagnosed and in 1.4%, the cause was unknown. The high percentage of undiagnosed patients probably represents most of the new patients interviewed, those whom had not been investigated. This finding is similar to a study carried out at the gynaecological clinic on etiological factors of infertility in the LUTH, where female factors accounted for 51.6%, whilst male factors were the sole findings in 21.6%. Combined factors accounted for the remaining 14.4%.^13^

Most of the respondents (69.1%) have been pregnant previously (secondary infertility), 30.9% have never been pregnant (primary infertility) which results in a ratio of approximately 1:2 between primary and secondary infertility. This is in contrast with a study conducted in the University of Ilorin Teaching Hospital (UITH), where it was found that 23.6% had primary infertility whilst 28.3% had secondary infertility (ratio of 1:1).^14^ The remaining 48.1% represented other gynaecological conditions. Amongst those who *have* been pregnant, only 27.7% had a child or children. The remaining 72.3% had pregnancies that probably resulted in spontaneous or induced abortions, still-births or peri-natal deaths. Amongst those who had children, 80.6% had one child, 17.9% had two children and 1.5% had more than two children.

Of the 350 respondents interviewed, 85.7% have heard of child adoption. This is similar to a study conducted in Eastern Nigeria where out of the 279 respondents, 86.4% had heard of child adoption.^5^ Amongst this group, the first major source of information about child adoption was the media (40.4%), closely followed by friends (32.3%), and then school (11.3%). Information from a doctor or nurse accounted for only 2%. Other sources included the church and people employed at orphanages.

Over half of the respondents (59.3%) knew the correct meaning of child adoption as a legal process of taking the child of another person as one's own, whilst (15.7%) thought that child adoption amounted to buying a motherless baby and 9.3% did not know what ‘adoption’ entailed. This differs from the findings of a similar study carried out in Enugu State, where only 27.3% of the 86.4% who were aware of child adoption, knew its correct meaning.^6^ About a quarter of the respondents (21.75%) knew the major requirements for adoption which is similar to a study where 21.6% of women knew how to adopt a baby in terms of meeting the requirements.^6^ The majority of the respondents (60.7%) did not know the maximum age of children who can be adopted, 12.0% thought that there was no maximum age and only 12.3% knew that the maximum age was 18 years. The majority (77%), however, knew the kind of children that could be adopted.

More than half of the respondents (68.3%) said that they could love an adopted child but less than half of them (33.7%) were willing to consider adoption. This disparity can probably be explained with ‘loving an adopted child is not the issue but blood ties [are]’ as seen in the major reason why respondents were not willing to adopt a child. In this group, only 9.7% of their spouse had considered adoption. This is similar to a national survey of more than 1400 randomly contacted adults in the United States which revealed that women were more likely than men to consider adoption, with a ratio of 42% to 35% respectively, as compared to this result of 33.7% to 9.7%.^15^ The major reason offered by the respondents for their unwillingness to adopt a child was that they desired to have their own biological child (82.6%). Of those willing to adopt a child, most (54.5%) would do so to rescue a child in an irreversible situation of abandonment and only 36.6% would adopt to ease the urge to care for a child. This is similar to a qualitative study on adoption practices amongst couples with secondary infertility in Karachi where less than half of the respondents (49.3%) admitted to ever considering adoption to cope with secondary infertility.^16^ It can therefore be reasoned that adoption amongst them is seen more as a humanitarian effort than a form of ‘treatment’ for infertility.

The preferred age for children to be adopted was new-born, that is, younger than six months (46.5%), and six months to two years (35.5%). Most of the respondents willing to adopt did have any particular gender preference (68.3%); however, a slight majority of the respondents would prefer to adopt a girl (18.8%) as opposed to a boy (12.9%). This is similar to the international and domestic adoption agencies who report a very strong preference for girls in the United States, regardless of whether the adopting couples are childless couples or individuals or whether they already have children.^17^ Just over half of the respondents willing to adopt (55.4%) felt children should be told they were adopted, whilst 44.6% felt that they should not be told. Most of the respondents (64.3%) felt that children should be told they were adopted only when they attain adulthood. There are two schools of thought regarding this, one that recommends ‘early’ sharing and one that believes in ‘late’ sharing. The first school of thought believes that if a child grows up with the word ‘adoption’ right from the start, he or she is familiar and comfortable with the situation of adoption and hence has no fear and doubts about their status. The latter school believes that the child has to be older and only after his cognitive development has occurred, would he or she be emotionally ready to accept the concept of adoption. The experience of most adoption agencies and adoptive parents has shown that ‘early sharing’ and ‘openness’ after taking into consideration the developmental level of the individual child, yields positive results.^18^

Of those who were willing to consider adoption, only 13.9% has adopted or attempted to adopt. A study conducted in Dublin, Ohio, revealed that although Americans are more positive about adoption than ever before, no more than 2% of the more than one-third who considered adopting, actually carried through with their intention.^15^

There was no statistically significant association between marriage type, age, religion, the duration of infertility and respondents’ willingness to adopt (*p* > 0.05). This is in contrast with a similar study conducted in Enugu where some factors associated with a favourable attitude towards adoption were, duration of infertility of longer than five years, no living child and a maternal age of more than 35 years^6^. Ethnicity, however, was statistically significant with regard to respondents’ willingness to adopt (*p* < 0.05) which revealed that Igbos were more willing to adopt than the Yorubas. This is similar to a past research study which showed evidence that most Nigerians, especially the Yorubas, have a negative attitude towards child adoption and adoptive parents.^19^ This is reflected in the stigmatisation amongst infertile women in the Yoruba tribe who are called ‘*agon*’, which is from a word that means to hold in contempt or to despise. This is probably a consequence of the societal belief that a woman has to prove her womanhood through motherhood and children are held as sources of pride, strength and economic fortune for the family.

There was also a statistically significant association between the respondents’ educational level and their willingness to adopt. It was shown that women of a higher educational level are more willing to adopt than women of lower educational level, probably because respondents with a higher educational level were more likely to know the correct meaning of child adoption. Respondents who knew the correct meaning of child adoption were more willing to adopt, which is similar to a study carried out in Enugu on child adoption where the correct meaning of child adoption was a significant predictor of consideration.^6^ Respondents whose spouses had considered child adoption were also more willing to consider it, showing that spousal opinion is a determinant factor in the respondent's willingness to adopt a child.

There was a statistically significant association between age, duration of infertility and respondents’ practice of child adoption (*p* < 0.05) with more women of an older age group (40–48 years) has either adopted or attempted to adopt, and more women with longer duration of infertility (more than 15 years) has either adopted or attempted to adopt. This is similar to a previous study where the percentage of adoption is high amongst couples married for 10 years or longer and especially in cases where the wife is more than 35 years old.^20^ There was no statistically significant association, however, between age and duration of infertility, to the respondent's attitude towards child adoption. This disparity can probably be related to the fact that respondents, who adopt a child, see it only as a last resort, a case of hopelessness or as a ‘*totka*’, meaning that when they adopt a child they could end up having their own child, and not because they actually want to adopt.

### Recommendations

Facility and community based health education programmes should be organised to educate couples and to encourage couples with infertility to adopt by highlighting its benefits and explaining the joys of parenting through adoption. The bond between a parent and child is one of ‘love’ that comes through ‘nurturing’ and not just the process of biological birth. This will help to educate couples and to improve their attitude with relation to the adopted and adoptees, thereby making adoption more acceptable to couples.

## Conclusion

This study investigated the knowledge, attitude and practice towards child adoption amongst women attending infertility clinics in teaching hospitals in Lagos State.

A high awareness of child adoption existed, but knowledge insufficient as less than a quarter of the participants knew the requirements for child adoption, the majority did not know the maximum age at which children could still be adopted and half of them do not know the requirements in terms of age for those that did want to adopt a child.

More than half of the respondents (68.3%) say they could love an adopted child but less than half of them (33.7%) were willing to consider adoption; furthermore, only 9.7% of their spouses have considered it. The urge for a biological child was the major reason for participants not willing to adopt a child. For the few that were willing to adopt, the preferred age was from new-born to two years with no particular gender preference (68.3%). Women of a higher educational level are more willing to adopt than women of a lower educational level.

## References

[1] Webster's unabridged dictionary http://www.answers.com/adoption#Websters_Unabridged_Dictionary_d.

[2] Benet MK. The politics of adoption New York: NY Free Press: New York, 1976; p.14.

[3] Boswell J. The kindness of strangers: The abandonment of children in Western Europe from late antiquity to the Renaissance Chicago: University of Chicago Press: Chicago, 1998; p. 62–63.

[4] Mrs Odukoya. Headquarters, Adoption Office, Social Welfare Department, Lagos State Ministry of Youths and Sports and Social Welfare, [Personal interview, n.d.] Alausa, Lagos Nigeria; n.y. (unpublished).

[5] Bhattachanya S. Infertility In: Edmonds DK, editor. Dewhurt's Textbook of Obstetrics and Gynaecology. 7th ed Oxford University Press: Blackwell Publishers, 2007; p. 440–460.

[6] Ezugwu FO, Obi SN, Onah HE. The knowledge, attitude and practice of child adoption among infertile Nigerian women. J Obstet Gynaecol. 2002;22(2):211–216. doi:10.1080/01443610120113463 12521712

[7] Pearce TO. She will not be listened to in public: Perceptions among the Yoruba of infertility and childlessness in women. Reprod Health Matter. 1999;7(13):69–78.

[8] Lagos State of Nigeria Official Gazette Child's Rights Law of Lagos State–A Law to enforce the Rights and advance the Welfare of the Child, and to amend and consolidate all legislations relating to the protection and welfare of the child in Lagos State and for other connected and incidental purposes, 2007; p. A314–A327.

[9] Araoye MO. Research Methodology with Statistics for Health and Social Sciences Nathdex Publishers: Ilorin Kwara State, Nigeria, 2004; p. 118.

[10] Dean AG, Burton AH, Dicker RC. Epi Info Version 6. A word processing, database and statistics program for epidemiology on microcomputers, USD Incorporated, Stone Mountain, Georgia; 1999.

[11] Obi SN. Extramarital sexual activity among infertile women in southeast [*sic*] Nigeria Department of Obstetrics and Gynecology, Federal Medical Centre, Abakaliki, Nigeria. J Obstet Gynecol India. 2006;56(1):72–75.

[12] Dyer SJ, Abrahams N, Mokoena NE, Van der Spuy ZM. ‘You are a man because you have children’: Experiences, reproductive health knowledge and treatment-seeking behaviour among men suffering from couple infertility in South Africa. Hum Reprod. 2004;19(4):960–967. doi:10.1093/humrep/deh195, PMid:15016772 15016772

[13] Giwa-Osagie OF, Ogumyeni D, Emuveyan EE, Akinla OA. Etiological classification and sociomedical characteristics of infertility in 250 couples. Int J Fertil. 1984;29:104–108. PMid:6148313 6148313

[14] Okonofua FE. Prevalence and risk factors for infertility in Southwest Nigeria In: Van Balen F, Gerrit T, Inhorn M, Social Science Research on Childlessness in a Global Perspective. Amsterdam, The Netherlands: SCO-Kohnstamm Institute; 2000. 11000715

[15] Evan Donaldson Institute National Adoption Attitudes Survey, June 2002; p. 20–38.

[16] Ali TS, Sami NS. Adoption practices among couples with secondary infertility in Karachi: A triangulation study design. JPMA. 2007;57(2):55–59. 17370784

[17] Charlene EM. The stigma of adoptive parent status: Perceptions of community attitudes toward adoption and the experience of informal social sanctioning. Fam Relat. 1987;36:34–39. doi:10.2307/584644

[18] Adamec C, Pierce W. The encyclopedia of adoption 2nd ed 2000; p. 256–70.

[19] Olubola T, Adejuwon G. Impact of self-esteem, locus of control and gender on attitude towards child adoption and adoptive parents among some adults in Ibadan metropolis. IFE PsychologIA. 2005;13(2):22–37.

[20] Sucharita P, Sayeed U. Involuntary childlessness: An exploratory study of men's perspective on infertility – A study of rural childless men in Andhra Pradesh in India. Int J Fertil. 1999;59:(2) 25–27.

